# Biogeography of anaerobic ammonia-oxidizing (anammox) bacteria

**DOI:** 10.3389/fmicb.2014.00399

**Published:** 2014-08-06

**Authors:** Puntipar Sonthiphand, Michael W. Hall, Josh D. Neufeld

**Affiliations:** Department of Biology, University of WaterlooWaterloo, ON, Canada

**Keywords:** anammox bacteria, biogeography, co-occurrence, 16S rRNA gene, brocadia, scalindua, planctomycetes

## Abstract

Anaerobic ammonia-oxidizing (anammox) bacteria are able to oxidize ammonia and reduce nitrite to produce N_2_ gas. After being discovered in a wastewater treatment plant (WWTP), anammox bacteria were subsequently characterized in natural environments, including marine, estuary, freshwater, and terrestrial habitats. Although anammox bacteria play an important role in removing fixed N from both engineered and natural ecosystems, broad scale anammox bacterial distributions have not yet been summarized. The objectives of this study were to explore global distributions and diversity of anammox bacteria and to identify factors that influence their biogeography. Over 6000 anammox 16S rRNA gene sequences from the public database were analyzed in this current study. Data ordinations indicated that salinity was an important factor governing anammox bacterial distributions, with distinct populations inhabiting natural and engineered ecosystems. Gene phylogenies and rarefaction analysis demonstrated that freshwater environments and the marine water column harbored the highest and the lowest diversity of anammox bacteria, respectively. Co-occurrence network analysis indicated that *Ca*. Scalindua strongly connected with other *Ca*. Scalindua taxa, whereas *Ca*. Brocadia co-occurred with taxa from both known and unknown anammox genera. Our survey provides a better understanding of ecological factors affecting anammox bacterial distributions and provides a comprehensive baseline for understanding the relationships among anammox communities in global environments.

## Introduction

The anaerobic ammonia oxidation (anammox) process converts ammonia to N_2_ gas by using nitrite as electron acceptor under anoxic conditions (van de Graaf et al., 1995). This process is important for removing fixed N from both engineered and natural systems and can be applied to wastewater treatment in order to replace conventional treatment systems. Anammox is cost effective and environmentally friendly because it does not require aeration or organic carbon inputs, and reduces the production of greenhouse gases (i.e., N_2_O and CO_2_) compared to conventional denitrification (Jetten et al., 1997; van Dongen et al., 2001); anammox was first implemented in a full-scale wastewater treatment plant (WWTP) in Rotterdam, Netherlands (van Dongen et al., 2001; Abma et al., 2007; van der Star et al., 2007). Although anammox bacteria were first discovered in WWTPs and their applications have been studied worldwide, they may account for more than 50% of N loss from marine environments (Arrigo, 2005; Francis et al., 2007). However, recent reports estimate that anammox bacteria contribute ~23–30% to N loss from marine environments (Trimmer and Engström, 2011; Dalsgaard et al., 2012; Babbin et al., 2014). The contributions of anammox bacteria to biogeochemical N_2_ production were measured as 18–36% in groundwater (Moore et al., 2011), 4–37% in paddy soils (Zhu et al., 2011), 9–13% in lakes (Schubert et al., 2006), and 1–8% in estuaries (Trimmer et al., 2003). These results indicate that anammox bacteria play a key role in the global N cycle.

Anammox bacteria branch deeply within the *Plantomycetes* phylum. There are five known anammox genera, with 16 species proposed to date. The first discovered anammox bacterium was *Ca*. Brocadia anammoxidans, enriched from a denitrifying fluidized bed reactor (Mulder et al., 1995; Kuenen and Jetten, 2001). The three characterized species within the *Ca*. Brocadia genera are *Ca*. Brocadia fulgida (Kartal et al., 2008), *Ca*. Brocadia sinica (Oshiki et al., 2011), and *Ca*. Brocadia caroliniensis (Rothrock et al., 2011); all of these were enriched in anammox bioreactors. The only species reported within the *Candidatus* Kuenenia genus is *Ca*. Kuenenia stuttgartiensis, which was isolated from a trickling filter biofilm (Schmid et al., 2000). The *Ca*. Scalindua genus consists of nine proposed species, six of which were discovered in marine environments (Kuypers et al., 2003; Woebken et al., 2008; Hong et al., 2011a; Fuchsman et al., 2012; Dang et al., 2013; van de Vossenberg et al., 2013). *Ca*. Scalindua sorokinii was the first anammox species found in a natural environment (the Black Sea; Kuypers et al., 2003). *Ca*. Scalindua richardsii was also recovered from the Black Sea (Fuchsman et al., 2012). Although these two species originated from the Black Sea, they dominated in different zones. A cluster associated with *Ca*. Scalindua sorokinii was detected in the lower suboxic zone where ammonium concentration was high, but nitrite concentration was low, whereas a cluster associated with *Ca*. Scalindua richardsii was found in the upper suboxic zone where ammonium concentration was low, but nitrite concentration was high (Fuchsman et al., 2012). *Ca*. Scalindua brodae and *Ca*. Scalindua wagneri were both identified in WWTPs (Schmid et al., 2003). *Ca*. Scalindua arabica originated in the Arabian Sea and the Peruvian oxygen minimum zone (OMZ; Woebken et al., 2008). *Ca*. Scalindua pacifica (Dang et al., 2013) and *Ca*. Scalindua profunda (van de Vossenberg et al., 2013) were retrieved from the Bohai Sea and a marine sediment of a Swedish fjord, respectively. Two additional species names were tentatively proposed from molecular surveys: *Ca*. Scalindua sinooilfield from a high temperature petroleum reservoir (Li et al., 2010) and *Ca*. Scalindua zhenghei from marine sediments (the South China Sea; Hong et al., 2011a). The only known species affiliated with the *Ca*. Anammoxoglobus genus was *Ca*. Anammoxoglobus propionicus, enriched from an anammox reactor (Kartal et al., 2007). *Ca*. Jettenia asiatica was retrieved from a granular sludge anammox reactor (Quan et al., 2008). Notably, known anammox bacteria species have mostly been discovered in engineered environments, but they have commonly been detected in various natural ecosystems and are more widespread than previously thought. However, it should be noted that *Ca*. Scalindua sinooilfield and *Ca*. Scalindua zhenghei are not in the category *Candidatus* on the list of prokaryotic names with standing in the nomenclature (LPSN) website. The classification and nomenclature of anammox *Ca*. species need to be better clarified and standardized in the future.

Observations of anammox bacterial diversity have demonstrated that *Ca*. Brocadia, *Ca*. Kuenenia, and *Ca*. Anammoxoglobus were commonly found in non-saline environments (i.e., Egli et al., 2001; Moore et al., 2011; Hu et al., 2013), whereas *Ca*. Scalindua dominated saline environments (i.e., Woebken et al., 2008; Hong et al., 2011a; Villanueva et al., 2014), including deep-sea methane seep sediments (Shao et al., 2014). Anammox bacteria have also been detected in extremely saline-related environments, including hydrothermal vents (Byrne et al., 2009; Russ et al., 2013), and cold hydrocarbon-rich seeps (Russ et al., 2013). However, because all previous molecular surveys of the anammox 16S rRNA genes were from individual studies of specific habitats, the overall understanding of global anammox bacterial diversities, distributions, and co-occurrences among lineages remains unclear.

Factors affecting anammox bacterial diversity and distribution have been investigated within individual habitat-specific studies. For example, organic carbon influenced anammox diversity in freshwater sediment (Hu et al., 2012b), soil (Shen et al., 2013), and an estuary (Hou et al., 2013). Ammonium and nitrite concentrations correlated with anammox diversity in a mangrove sediment (Li et al., 2011). Temperature impacted anammox communities in freshwater sediment (Osaka et al., 2012) and an estuary (Hou et al., 2013). Depth affected anammox diversity in marine sediment (Li et al., 2013). However, no comprehensive survey has previously explored factors that govern global anammox distributions.

The main objectives of this study were to investigate global anammox bacterial distributions and identify factors influencing anammox bacterial distributions and diversity. Over 6000 anammox 16S rRNA gene sequences from Genbank were collected and analyzed by both phylogenetic and multivariate statistical methods. An anammox 16S rRNA gene phylogenetic tree revealed broad anammox distributions across habitats, including marine sediment, marine water column, estuary, mangrove sediment, soil, freshwater, freshwater sediment, groundwater, reactor, WWTP, marine sponge, biofilter, fish gut, shrimp pond, and oil field. Co-occurrence analysis demonstrated strong relationships among dominant anammox phylotypes. Global distributions of anammox bacteria revealed factors that influence anammox bacterial distributions, with salinity being the most important environmental variable. This study provides a better understanding of the prevalence of anammox bacterial 16S rRNA genes across habitats and the key factors impacting their distribution patterns.

## Materials and methods

### Data collection and preparation

All anammox 16S rRNA gene sequences available in Genbank were extracted on October 25th, 2013. In total, 14,790 potential anammox-related sequences were collected using the following keyword searches: “*uncultured planctomycete 16S ribosomal RNA gene*,” “*anammox bacterium 16S ribosomal RNA gene*,” “*anaerobic ammonium-oxidizing bacterium 16S ribosomal RNA gene*,” “*Candidatus Brocadia 16S ribosomal RNA gene*,” “*Candidatus Scalindua 16S ribosomal RNA gene*,” “*Candidatus Kuenenia 16S ribosomal RNA gene*,” “*Candidatus Anammoxoglobus 16S ribosomal RNA gene*,” and “*Candidatus Jettenia 16S ribosomal RNA gene*.” Most anammox bacterial 16S rRNA gene sequences were deposited in the Genbank with the definition “*uncultured planctomycete 16S ribosomal RNA gene*” (data not shown). However, this keyword-based search retrieved both anammox and non-anammox sequences. All collected sequences were searched by BLAST against known anammox species in Genbank core reference set and aligned by QIIME v1.7 (Caporaso et al., 2010) using Infernal (Nawrocki and Eddy, 2013) against the Greengenes database (May 2013 revision; DeSantis et al., 2006) to screen for anammox-related sequences. After removing non-anammox and low quality sequences, over 6000 sequences from >200 isolation sources were included in the analysis. All anammox sequences from across many specific “Isolation source” Genbank designations were assigned to 15 general habitats: marine sediment, marine water column, estuary, freshwater sediment, freshwater, groundwater, soil, mangrove sediment, WWTP, reactor, marine sponge, biofilter, fish gut, oil field, and shrimp pond.

Limitations of this analysis included metadata inconsistencies and missing environmental parameters across multiple studies. Consequently, metadata were qualitatively grouped into three broad categories: salinity (saline, mixed, and non-saline environments), ecosystem (natural and engineered), and habitat (listed above). Another limitation was that it was not possible to consistently determine relative abundances of anammox sequences within each study due to inconsistencies with reporting, sampling efforts, and methodologies. To address this shortcoming, all anammox 16S rRNA gene sequences were clustered into operational taxonomic units (OTUs) at 97% identity with cd-hit-est v4.5.4 (Fu et al., 2012) and the abundance of each anammox OTU was only counted as present or absent for each study.

### Statistical and multivariate analyses

Individual studies that contributed anammox 16S rRNA gene sequences were usually associated with unique Genbank isolation sources. Because of this, the numbers of anammox 16S rRNA gene sequences contributed per study and/or unique isolation source were broad, ranging from 1 to 623 sequences. In order to ensure that dissimilarity matrices were generated from datasets derived from the same number of sequences from each study, multiple rarefied datasets were generated that varied in the number of sequences derived from each study/isolation source. In cases where multiple studies represented compatible isolation sources, yet with relatively low numbers of sequences, these sequence data were pooled into additional isolation source categories to maximize habitat representation in the rarefied analyses. Subsequently, we tested datasets rarefied to 10, 40, or 100 sequences from each isolation source category.

After clustering the sequences at 97% identity, all sequences were aligned and trimmed in order to consider a single homologous spanning region of the 16S rRNA gene, which corresponded to the positions 384–834 of *Escherichia coli* (J01695.2; Brosius et al., 1978). Any sequences with less than 100 bases after trimming were discarded from the analysis. Consequently, the sequences from some isolation sources within five minor habitats (marine sponge, biofilter, fish gut, oil field, and shrimp pond) fell below the threshold for rarefied datasets 40 and 100 sequences. All five of these minor habitats were removed from further analysis. The minimum sequence threshold remained at 10, 40, and 100 after being trimmed. Consequently, 10 major habitats (marine sediment, marine water column, estuary, freshwater sediment, freshwater, groundwater, soil, mangrove sediment, WWTP, reactor) were considered in this analysis.

Principal coordinates analysis (PCoA) ordinations were generated from unweighted UniFrac distance matrices (Lozupone and Knight, 2005) through QIIME (Caporaso et al., 2010). Non-metric multidimensional scaling (NMDS) ordinations were calculated based on a Jaccard dissimilarity matric, using the AXIOME pipeline (Lynch et al., 2013). To test treatment effects and within-group agreement, multi-response permutation procedures (MRPP) were tested on 999 permutations, using the R library *vegan* (Oksanen et al., 2008) from within AXIOME. Analyzed data for each rarefied dataset (10, 40, and 100 sequences), including the OTU table with taxonomic classifications and the analyzed sequences, a mapping file, and the source FASTA files, are in a single compressed Supplementary Material file (“Sonthiphand supp data files.zip”). All collected sequences, with corresponding Genbank accession numbers and metadata, are provided in a spreadsheet (sequences.xlsx) within the Supplementary Material.

### Rarefaction curve and diversity indices

Rarefaction curves, observed species, phylogenetic diversity (PD), Chao1, and Shannon indices were generated by QIIME (Caporaso et al., 2010). The Wilcoxon Signed-rank test was performed by the R function *wilcox.test* (R Core Team, 2013). The null hypothesis was that the number of OTUs between habitats was the same. If *p* was ≤ 0.05, the null hypothesis was rejected.

### Phylogenetic construction

Representative sequences for each OTU from each habitat were selected for phylogenetic analysis. A total of 505 OTU sequences from across all 15 habitats included all know anammox *Candidatus* species. Outgroups included cultured non-anammox species of *Planctomycetales*, including *Planctomyces maris* (X62910), *Isophaera* sp. (X81958), *Gemmata obscuriglobus* (X85248), *Blastopirellula marina* (HE861893), *Rhodopirellula baltica* (FJ624346), and *Pirellula* sp. (X81942). Sequences were aligned using MUSCLE (Edgar, 2004) and trimmed to a final homologous length of ~310 bases. A maximum likelihood tree was constructed with the PhyML v.3.0.1, using the GTR model (Guindon and Gascuel, 2003). The tree topology was optimized at five random starts. The approximate likelihood ratio test (aLRT) was conducted to provide tree topology support. The phylogenetic tree was visualized by SEAVIEW (Galtier et al., 1996).

### Co-occurrence network analysis

Anammox sequences were sorted by habitat and an OTU table was generated by AXIOME. Co-occurrence was assessed using a previously described method (Barberán et al., 2012). All singletons were discarded, and OTUs having a Spearman's correlation ≥ 0.8 were considered to have a strong co-occurrence relationship. Spearman's correlation was used because it only checks if two OTUs are monotonically related, rather than having a linear relationship. As a result, it is less sensitive to differences in abundance, and this was desirable because abundance information may have been lost when the sequences were deposited in GenBank, as described above. The results were visualized with Gephi (Bastian et al., 2009).

## Results

### Distributions of anammox bacteria across habitats

**Figure 1:**
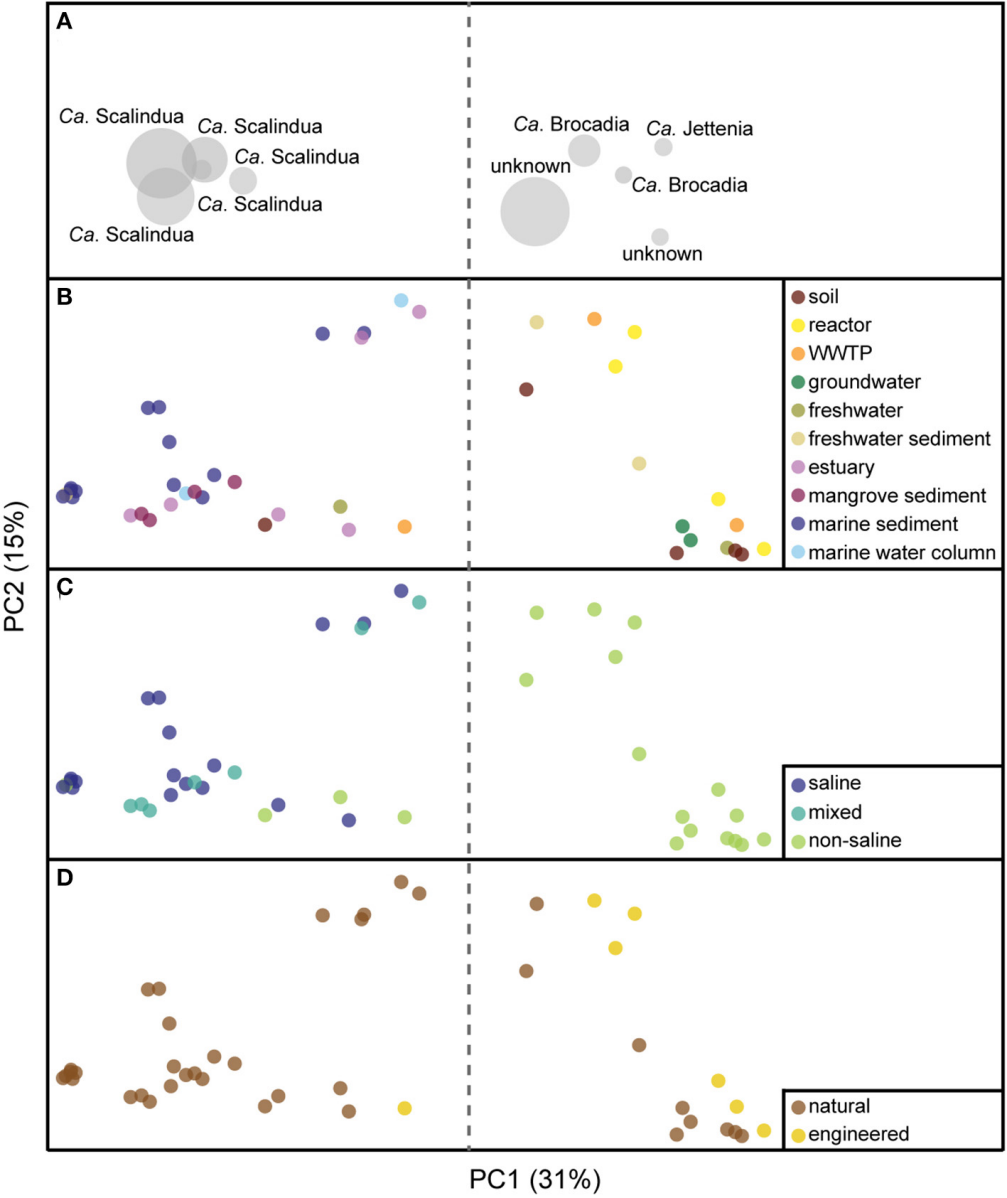
**Principal coordinate analysis (PCoA) ordination based on an unweighted UniFrac distance matrix of anammox bacterial 16S rRNA gene profiles**. The taxonomic biplot information for all panels is represented in **(A)**. Panels **(B–D)** show distributions of anammox isolation source representation (points) colored by habitat, salinity, and ecosystem, respectively. The proportion of the variation explained is indicated on the axes.

**Figure 2:**
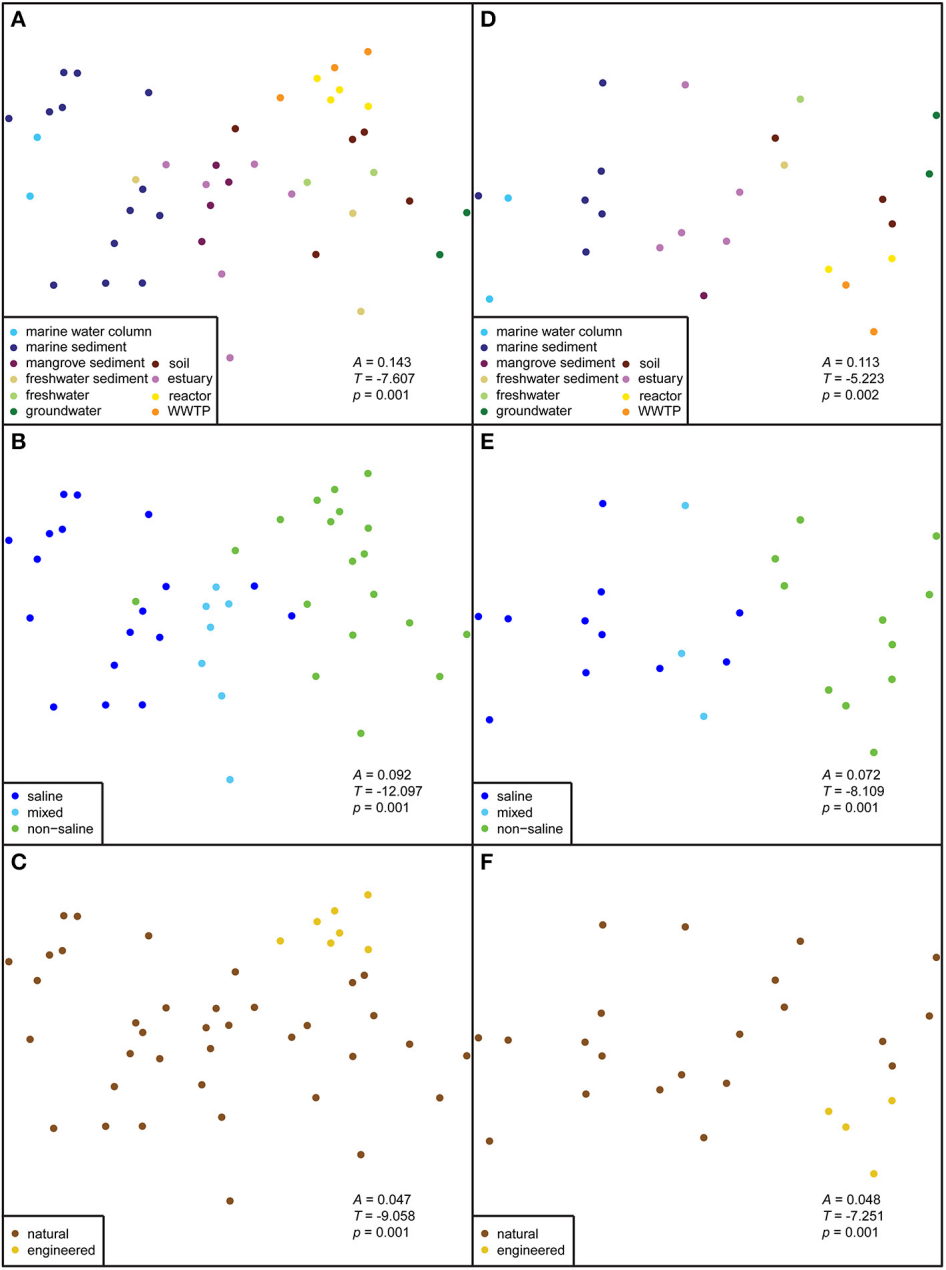
**Non-metric multidimensional scaling (NMDS) plots of anammox 16S rRNA gene sequences**. The correlations of habitat, salinity, and ecosystem were calculated by a Jaccard dissimilarity metric. The first column **(A–C)** shows the ordination results for datasets rarefied to 40 sequences; all sequences were from 44 isolation sources. The second column **(D–F)** shows the datasets rarefied to 100 sequences; all sequences were from 25 isolation sources. The significance of group separations (*A, T*, and *p*) are indicated within each ordination.

Anammox sequences were collected from multiple studies and isolation sources. The number of sequences was considerably different from one isolation source to another. Three rarefied sequence collections were generated to compare distribution patterns. Because the broad range of analyzed sequences (10–623 sequences) affected dissimilarity measurements, we chose to analyze set 40 in more detail to include as many isolation sources as possible in our analysis while maximizing sequence sample size (Figure 1). This was done because set 10 (i.e., 10 sequences per isolation source) showed poor groupings with low correlations (data not shown) and both set 40 and set 100 showed similar distribution patterns with high correlations (Figure 2).

All anammox sequences from 10 habitats were visualized within an ordination plot based on phylogenetic distances by using an unweighted UniFrac distance matrix (Figure 1). The percentage of PCoA principal coordinates (PC1 and PC2) explained 46% variability among all samples. The ordination demonstrated that anammox sequences clustered significantly by habitat (Figure 1B), which was supported by MRPP (*T* = −7.6, *A* = 0.14, *p* < 0.001; Figure 2). All anammox sequences clustered separately into two main groups (Figure 1B). Marine sediment, marine water column, estuary, and mangrove sediment grouped together and were dominated by *Ca*. Scalindua cluster (Figures 1A,B). The WWTP, reactor, soil, freshwater, freshwater sediment, and groundwater grouped together and were dominated by *Ca*. Brocadia, *Ca*. Jettenia, and the unknown cluster. Four samples, one each from freshwater, freshwater sediment, soil, and WWTP, were present in both groups.

### Key factors affecting global anammox bacterial distribution

The strongest separation of anammox bacterial sequences was linked to sample salinity (Figure 1C), which we assigned qualitatively as saline, “mixed,” and non-saline environments. The mixed environments were generally river-marine transitional zones, mostly from mangrove and estuary habitats. Saline and mixed environments clustered together and differed significantly from non-saline environment (Figures 1C, 2B; *T* = −12.1, *A* = 0.09, *p* < 0.001). However, a few non-saline samples grouped with saline and mixed samples. The *Ca*. Scalindua cluster was clearly dominant in saline environments but almost never detected in non-saline environments (Figures 1A,C). The major complement of anammox bacteria found in non-saline environment was *Ca*. Brocadia, *Ca*. Jettenia, and the unknown clusters. The results indicated that salinity was the key factor governing global distributions of anammox bacteria.

### Distinct anammox bacteria in natural and engineered ecosystems

Another factor that showed a significant correlation with the anammox bacterial distributions was ecosystem type. Although most anammox sequences were from natural ecosystems, those from engineered ecosystems grouped together (Figures 1D, 2C; *T* = −9.1, *A* = 0.05, *p* < 0.001). However, one sample from a WWTP grouped separately from other samples of engineered ecosystems (Figure 1D). This WWTP sample contained very few anammox sequences associated with *Ca*. Scalindua cluster. More robust group separation was visualized by the NMDS generated from an OTU-based Jaccard distance metric (Figures 2C,F). This observation demonstrated environmental selection of anammox bacteria in natural and engineered ecosystems.

### Diversity richness of anammox bacteria

**Figure 3:**
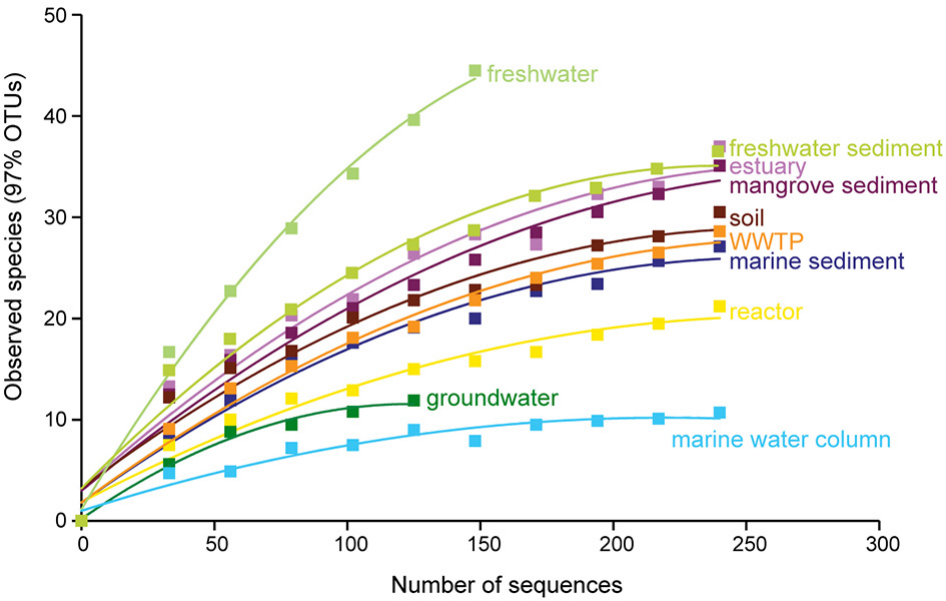
**Rarefaction curves of the anammox bacterial 16S rRNA gene diversity among sampled habitats**. OTUs were generated at 97% identity. The *p*-values for differences among all habitats are shown in Table 2.

Rarefaction curves and diversity indices showed that freshwater possessed the highest anammox bacterial diversity, whereas the marine water column was associated with the lowest diversity (Figure 3 and Table 1). The diversity of anammox bacteria in freshwater and marine water column differed significantly (*p* = 0.01). The diversity of anammox bacteria in freshwater and freshwater sediment was not significantly different (*p* = 0.22). Rarefaction curves of freshwater showed no saturation, although only 170 sequences were analyzed. The majority of freshwater anammox sequences were from unpublished data; only a few publications reported anammox bacterial 16S rRNA gene sequences from freshwater (Schubert et al., 2006; Hamersley et al., 2009; Pollet et al., 2011; Han and Gu, 2013; Sonthiphand and Neufeld, 2013). Consequently, more research on anammox bacteria in freshwater would be required to confirm this observation. Overall, the results imply that most novel anammox clusters remain undiscovered within freshwater habitats.

**Table 1:** **The number of anammox sequences and diversity indices for each habitat**.

**Habitat**	**Total collected sequences**	**Total analyzed sequences**	**Diversity indices**		
			**PD**	**Chao1**	**Shannon**
Marine sediment	2046	1921	1.11	35.2	2.66
Marine water column	325	324	0.74	10.3	1.07
Estuary	1365	1347	0.99	53.3	3.66
Freshwater sediment	479	473	1.64	35.5	3.59
Freshwater	170	170	2.26	103.5	3.93
Groundwater	472	126	0.55	13.7	2.06
Soil	816	815	0.78	28.0	3.33
Mangrove sediment	366	339	1.22	42.4	3.30
WWTP	288	249	1.30	36.3	2.77
Reactor	420	355	1.10	22.9	2.51

*PD, Phylogenetic diversity*.

The diversity of anammox bacteria in marine sediments was higher than in the marine water columns (*p* = 0.02; Figure 3, Table 1). The reason for this observation might be higher physical and biogeochemical heterogeneity in marine sediments, associated with a greater overall microbial diversity (Table 1). The diversity of anammox bacteria among other isolation source samples, including freshwater sediment, estuary, mangrove sediment, soil, and marine sediment, showed no significant differences (Figure 3, Table 2). The diversity of anammox bacteria in engineered ecosystems, including WWTPs and reactors, were not significantly different (*p* = 0.15), consistent with the observation that anammox bacteria from engineered ecosystems grouped together (Figures 1D, 2C,F).

**Table 2:** **Significance of richness differences among 10 habitats, calculated by the Wilcoxon Signed-rank test**.

**Habitat**	**Estuary**	**Freshwater**	**Freshwater sediment**	**Groundwater**	**Mangrove sediment**	**Marine sediment**	**Marine water column**	**Reactor**	**Soil**
Freshwater	0.10	−	−	−	−	−	−	−	−
Freshwater sediment	0.69	0.22	−	−	−	−	−	−	−
Groundwater	**0.01**	**0.01**	**0.01**	−	−	−	−	−	−
Mangrove sediment	0.69	0.10	0.55	**0.01**	−	−	−	−	−
Marine sediment	0.15	0.03	0.10	0.12	0.31	−	−	−	−
Marine water column	**0.01**	0.01	**0.01**	0.10	**0.01**	**0.02**	−	−	−
Reactor	0.02	0.01	**0.02**	0.22	**0.03**	0.31	**0.03**	−	−
Soil	0.42	0.06	0.31	**0.01**	0.69	0.42	**0.01**	**0.03**	−
WWTP	0.15	**0.03**	0.15	0.06	0.31	0.84	**0.01**	0.15	0.55

*Significant differences (p < 0.05) are shown in bold*.

Although groundwater, freshwater, and freshwater sediment were non-saline isolation sources, the diversity of groundwater was low and significantly different from freshwater (*p* = 0.01) and freshwater sediment (*p* = 0.01; Table 1). However, the interpretation of this observation must be cautious because only a few publications have surveyed anammox bacterial 16S rRNA gene sequences in groundwater (Hirsch et al., 2011; Moore et al., 2011; Sonthiphand and Neufeld, 2013). Only 126 sequences were included in this analysis; however, 472 anammox sequences were collected from Genbank (Table 1). The majority of groundwater anammox sequences were from contaminated groundwater in Canada (Moore et al., 2011), and most sequences were excluded due to the region of analyzed 16S rRNA genes being outside of the region used to generate a phylogenetic tree, which was the basis of this analysis.

### Phylogeny and co-occurrence of anammox bacteria

**Figure 4:**
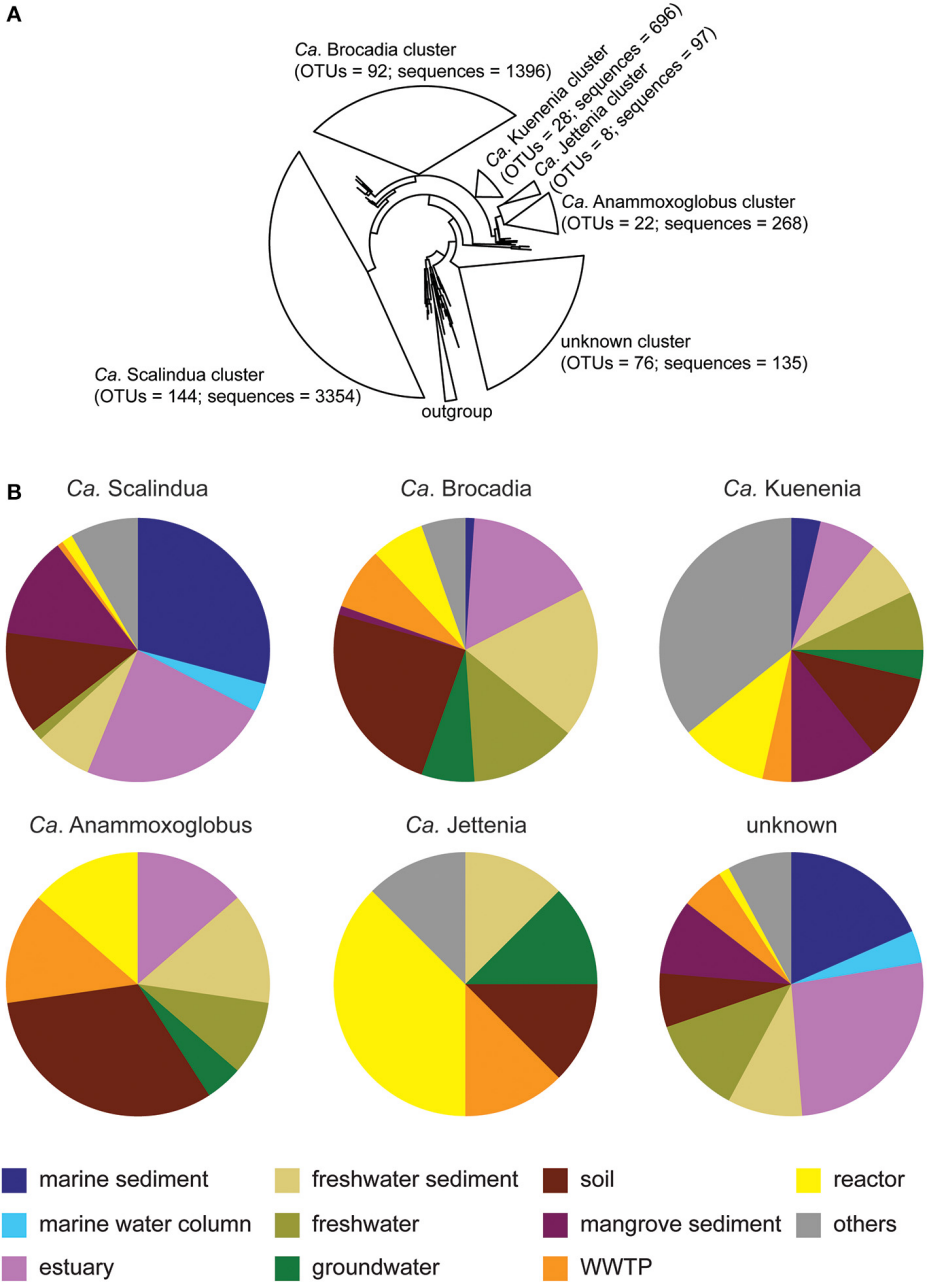
**Phylogeny and composition of anammox bacteria**. **(A)** A 16S rRNA based-phylogenetic tree of representative anammox OTU sequences from 15 habitats: marine sediment, marine water column, estuary, freshwater, freshwater sediment, groundwater, reactor, WWTP, marine sponge, biofilter, fish gut, shrimp pond, and oil field. The OTUs were generated at 97% identity. The OTU sequences grouped into five known anammox clusters and one unknown cluster. The numbers of OTUs and anammox sequences were shown in the bracket of each cluster. **(B)** Annotated habitat representation within six anammox clusters. “Others” represent five minor habitats, including marine sponge, biofilter, fish gut, shrimp pond, and oil field.

The dominant anammox phylotypes recovered from across all isolation sources were *Ca*. Scalindua and *Ca*. Brocadia, in addition to lower abundance anammox phylotypes, including *Ca*. Kuenenia, *Ca*. Anammoxoglobus, and *Ca*. Jettenia (Figure 4A). The unknown cluster comprised of 76 OTUs; however, the average sequences per OTU were only 1.78 sequences. There was no majority of anammox sequences per OTU for the unknown cluster, reflecting that the unknown anammox clusters were likely low abundance but high diversity anammox bacteria, possibly representing part of the rare biosphere of these isolation sources.

Approximately 70% of total *Ca*. Scalindua OTU sequences were from saline-related environments, including marine sediment, marine water column, estuary, and mangrove sediment (Figure 4B). *Ca*. Scalindua was also detectable in soil and freshwater-related environments, representing 13 and 8% of all anammox OTUs from those isolation sources, respectively.

*Ca*. Brocadia was most commonly retrieved from non-saline environments, including freshwater sediment, freshwater, groundwater, and soil (Figure 4B). All freshwater-related environments and soil accounted for 38 and 24% of *Ca*. Brocadia OTU sequences, respectively. Engineered ecosystems, including WWTP and reactor, accounted for 15% of *Ca*. Brocadia OTU sequences. Although 16% of *Ca*. Brocadia OTU sequences were recovered from estuary isolation sources, only 1% of these OTUs were associated with marine sediment (Figure 4B). No *Ca*. Brocadia sequences were detected in marine water column data.

*Ca*. Kuenenia was the third most abundant cluster found across all isolation sources (Figure 4A). This cluster was detected across nine of the main habitats, but not the marine water column (Figure 4B). *Ca*. Kuenenia was also found in all five minor habitats, including marine sponge, biofilter, fish gut, shrimp pond, and oil field. Although *Ca*. Kuenenia was present in almost all habitats, a few OTUs (1–3 OTUs) per habitat were discovered. This observation indicated that *Ca*. Kuenenia cluster was not ubiquitous, but still widespread across habitats.

The *Ca*. Anammoxoglobus cluster was distributed similarly to the *Ca*. Brocadia cluster across isolation sources. For example, soil and freshwater-related environments accounted for 32 and 28% *Ca*. Anammoxoglobus OTU sequences (Figure 4B), respectively (compare to 24 and 38% for *Ca*. Brocadia, respectively). Estuary, WWTP, and reactor equally accounted for 14% of total *Ca*. Anammoxoglobus OTUs. Marine sediment and marine water column samples did not contribute OTUs from the *Ca*. Anammoxoglobus cluster.

The lowest abundance of known anammox bacterial genera was *Ca*. Jettenia, which comprised only eight OTUs (Figure 4A). Although *Ca*. Jettenia was not commonly detected within most isolation sources, the majority of this cluster was retrieved from engineered ecosystems, including WWTPs and reactors (Figure 4B). These engineered isolation sources accounted for 51% of all recovered *Ca*. Jettenia OTUs. Freshwater sediment, groundwater, and soil equally accounted for 13% of total *Ca*. Jettenia OTUs. None of *Ca*. Jettenia OTUs were associated with saline-related environments (Figure 4B).

The distributions of anammox bacterial OTUs of the unknown cluster were relatively similar to those of the *Ca*. Scalindua cluster (Figure 4B). The majority of sequences found in this cluster was from saline environments, including marine sediment, marine water column, estuary, and mangrove sediment; they accounted for 57% of the unknown OTU sequences. Freshwater, freshwater sediment, soil, and WWTPs accounted for 12, 9, 7, and 5% of unknown OTU sequences, respectively. As with the *Ca*. Scalindua cluster, the unknown cluster was present across nine of the main habitats, but not found in groundwater.

**Figure 5:**
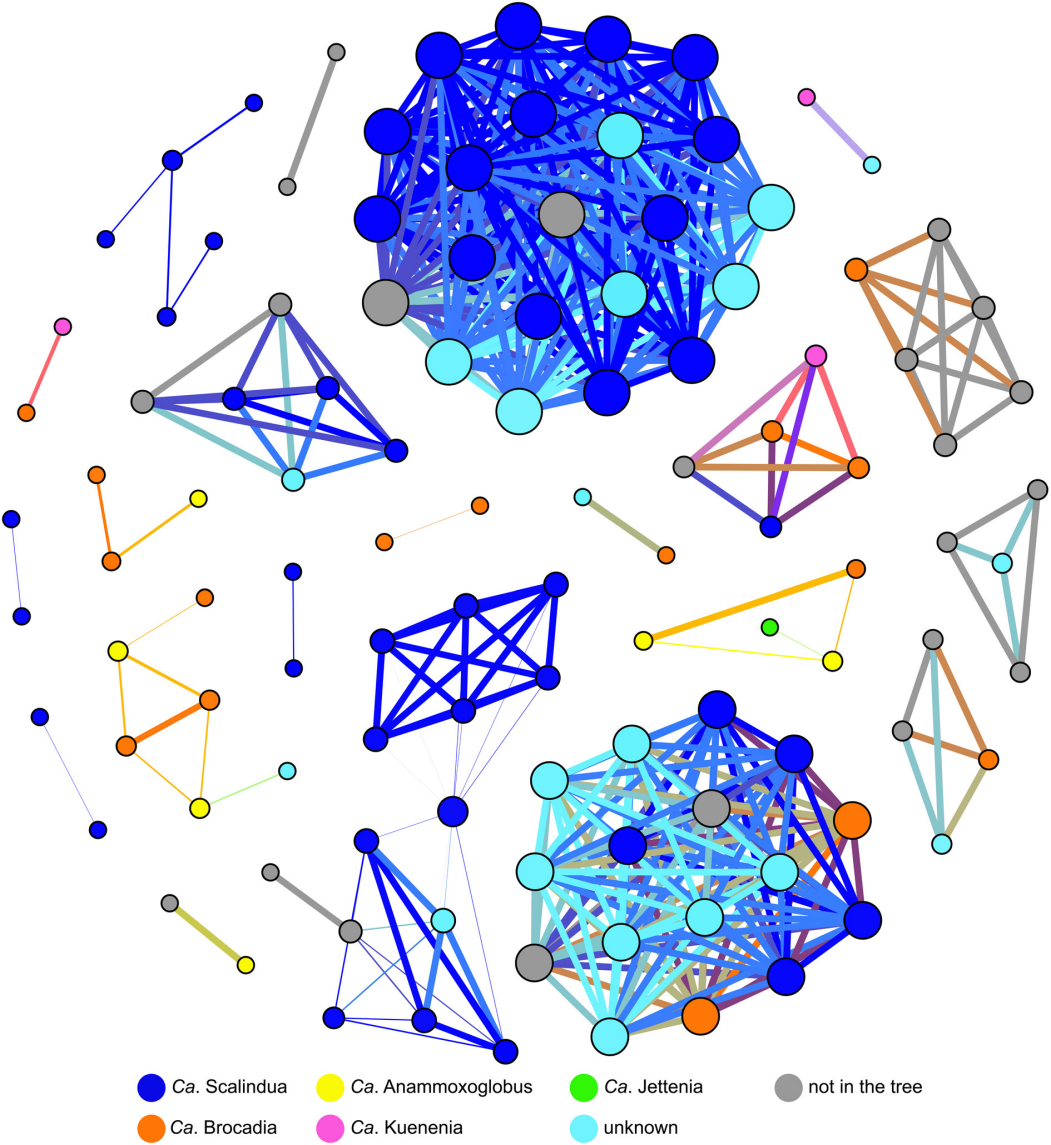
**Co-occurrence network of 97% OTU identity sequences from 15 habitats**. A connection represents a strong correlation (Spearman's ≥ 0.8). Singleton OTU sequences were removed. Nodes are colored by anammox cluster according to a phylogenetic tree (Figure 4A). Some of the OTUs were excluded from the network prior to the analysis because of differing 16S rRNA gene regions contained within the analysis. Node sizes represent the number of connections and edge width represents correlation strength.

Co-occurrence patterns suggested that *Ca*. Scalindua OTUs correlated very well with other *Ca*. Scalindua OTUs (Figure 5). In some cases, *Ca*. Scalindua was found together with *Ca*. Brocadia, *Ca*. Kuenenia, and OTUs from the additional unknown cluster. Strong co-occurrences of *Ca*. Scalindua with *Ca*. Anammoxoglobus and *Ca*. Jettenia were not observed. *Ca*. Brocadia OTUs within the co-occurrence network were correlated with OTUs spanning all known genera and the unknown anammox cluster (Figure 5). *Ca*. Anammoxoglobus correlated consistently with *Ca*. Brocadia, indicating a close relationship between OTUs of these two genera. Although eight OTUs of *Ca*. Jettenia were reported (Figure 4A), singleton OTUs were removed from this network analysis. Only one main *Ca*. Jettenia OTU formed part of a co-occurrence network (Figure 5). A *Ca*. Jettenia OTU correlated with a *Ca*. Anammoxoglobus OTU, and these linked to a *Ca*. Brocadia OTU. Overall, the resulting network revealed the close relationships among OTUs of *Ca*. Jettenia, *Ca*. Anammoxoglobus, and *Ca*. Brocadia clusters. The closest co-occurring genus to *Ca*. Kuenenia was *Ca*. Brocadia (Figure 5). The co-occurrence of *Ca*. Kuenenia with *Ca*. Scalindua and one OTU of the unknown cluster was also observed.

## Discussion

Based on an ordination analysis and a non-parametric analysis of the distance matrix, we confirmed that salinity is the dominant factor governing the global distribution of anammox bacteria (Figures 1A,C). These results are not surprising given that within-study correlation analyses have previously demonstrated that salinity influenced the geographical distribution of anammox bacteria in estuary and marsh sediments (Dale et al., 2009; Hu et al., 2012a; Hou et al., 2013). *Ca*. Scalindua dominated saline environments, including marine sediment, marine water column, estuary, and mangrove sediment. The comprehensive phylogenetic analysis also supported that ~70% of *Ca*. Scalindua were from saline environments (Figures 4A,B). These results are consistent with previous observations that a lab-scale bioreactor community dominated by *Ca*. Kuenenia shifted toward *Ca*. Scalindua dominance after being enriched in high salt concentrations for 360 days (Kartal et al., 2006). In addition, salinity showed negative correlations with *Ca*. Scalindua diversity in the Bohai Sea sediment (Dang et al., 2013), which would be consistent with the low overall diversity we observed for saline environments surveyed here (Figure 3).

Although there is no pure anammox culture available so far, comparative metagenomic studies of *Ca*. Kuenenia (Strous et al., 2006; Speth et al., 2012), *Ca*. Brocadia (Gori et al., 2011), *Ca*. Jettenia (Hu et al., 2012c), and *Ca*. Scalindua (van de Vossenberg et al., 2013; Villanueva et al., 2014) revealed that *Ca*. Scalindua has unique characteristics that support marine environment adaptations. *Ca*. Scalindua has high-affinity ammonium transport (*amtB*) and formate/nitrite transport (*focA*) proteins; both genes are highly expressed compared to those present in other anammox species (van de Vossenberg et al., 2013). These characteristics help *Ca*. Scalindua adapt to marine environments where ammonium and nitrite may be limited (Lam and Kuypers, 2011). So far, only *Ca*. Scalindua is known to contain genes involved in dipeptide and oligopeptide transport with moderate expression (van de Vossenberg et al., 2013). Consequently, *Ca*. Scalindua has an alternative ammonium source from degraded and mineralized organic matter. *Ca*. Scalindua also has a relatively versatile metabolism. *Ca*. Scalindua can use NO^−^_2_, NO^−^_3_, and metal oxides as alternative electron acceptors (van de Vossenberg et al., 2008, 2013). In the presence of organic acids (i.e., propionate, acetate, formate), *Ca*. Scalindua can perform dissimilatory nitrate reduction to ammonia (DNRA; Jensen et al., 2011). Lipid assays demonstrated that ladderane lipids with three cyclobutane rings and one cyclohexane ring may be specific to *Ca*. Scalindua (Kuypers et al., 2003; Kuypers et al., 2005; van de Vossenberg et al., 2008). However, this unique lipid structure may or may not facilitate *Ca*. Scalindua being dominant in marine environments. The specific function of this lipid needs further biochemical assays to verify.

Previous research demonstrates that salinity impacts not only the distribution patterns and diversity of anammox bacteria but also their abundance and activity. The abundance of anammox bacteria increased with the salinity gradients in Cape Fear River estuary (Dale et al., 2009) and Yangtze estuary (Hou et al., 2013). In contrast to their abundance, the activity of anammox bacteria was negatively correlated with salinity (Trimmer et al., 2003; Rich et al., 2008; Koop-Jakobsen and Giblin, 2009). However, salinity can be linked with other factors such as NO^−^_3_, NH^+^_4_, vegetation zones, and relative contribution to denitrifiers; it can be difficult to confirm the independent effect of salinity on anammox activity (Koop-Jakobsen and Giblin, 2009).

The links between salinity and anammox bacterial distributions are also more broadly observed for other microorganisms within a broad range of habitats. For example, the abundance and diversity of ammonia oxidizing bacteria (AOB) and archaea (AOA) were affected by salinity (Francis et al., 2003; Santoro et al., 2008; Biller et al., 2012). The diversity of denitrifying bacteria in WWTP systems was affected by salinity (Yoshie et al., 2004) and inhibitory effects of salinity on nitrification and denitrification rates were observed in estuary sediment (Rysgaard et al., 1999). Not only does salinity affect the distributions of specific groups of microorganisms, salinity impacted community fingerprints and species richness estimates for Bacteria, Archaea, and Eukaryotes within a solar saltern in Spain (Casamayor et al., 2002). The bacterial community composition along an estuary shifted due to a salinity gradient (Crump et al., 2004). Statistical and multivariate approaches have also confirmed salinity as the key factor driving global distribution patterns of Bacteria (Lozupone and Knight, 2007) and Archaea (Auguet et al., 2010).

Ordinations, including PCoA (Figure 1D) and NMDS (Figures 2C,F), showed that anammox bacteria from natural ecosystems formed clusters apart from those of engineered ecosystems. This observation suggests environmental selection of anammox bacteria in natural and engineered ecosystems. Reasons for this finding include differences in the physiological properties of anammox bacteria, including specific growth rate (μ_*max*_), affinity for ammonia and nitrite (K_*s*_), optimum growth temperature, and pH. The physiological properties of *Ca*. Kuenenia stuttgartiensis (Egli et al., 2001; van der Star et al., 2008a,b), *Ca*. Brocadia anammoxidans (Strous et al., 1998, 1999; Jetten et al., 2005), and *Ca*. Brocadia sinica (Oshiki et al., 2011) are now characterized. These physiological properties demonstrate that *Ca*. Brocadia sinica adapts better to engineered ecosystems because of a lower affinity for ammonia and nitrite, higher tolerance to O_2_, and higher growth rate (Oshiki et al., 2011). Engineered ecosystems are typically associated with high ammonia and nitrite loads. Wastewater treatment technologies apply O_2_ to facilitate AOB activity so that the coexistence of anammox bacteria and AOB transforms fixed N to N_2_ gas (Third et al., 2001; van Dongen et al., 2001).

After being enriched in fluctuating nitrite concentrations, a *Ca*. Brocadia dominated community shifted to a *Ca*. Kuenenia dominated community due to differences in affinity for NO^−^_2_ (van der Star et al., 2008a). *Ca*. Scalindua from marine environment changed to *Ca*. Brocadia and *Ca*. Kuenenia after being enriched in a bioreactor (Nakajima et al., 2008). Either *Ca*. Brocadia or *Ca*. Kuenenia was commonly dominant in lab-scale bioreactors (Egli et al., 2001; Hu et al., 2010; Park et al., 2010). In this study, network co-occurrence analysis showed that *Ca*. Brocadia and *Ca*. Kuenenia OTUs are correlated with one other (Figure 5). However, more research on physiological properties, including kinetic and biochemical analyses, of other anammox species are needed to better understand niche differentiation of anammox bacteria in different ecosystems.

Although the diversity of anammox sequences from marine water column and marine sediment was significantly different (Figure 3, Table 2), marine environments harbored a low overall diversity of anammox bacteria, mostly restricted to *Ca*. Scalindua (i.e., Schmid et al., 2007; Woebken et al., 2008; Hong et al., 2011a,b). A microdiversity within *Ca*. Scalindua was previously discovered in marine OMZs, comprising several subclusters (Woebken et al., 2008). The microdiversity of *Ca*. Scalindua was also found in other marine environments, including the South China Sea (Hong et al., 2011a; Han and Gu, 2013), the Jiaozhou Bay (Dang et al., 2010), the Bohai Sea (Dang et al., 2013), the Columbian Pacific (Castro-González et al., 2014), and deep-sea methane seep sediments in the Okhotsk Sea (Shao et al., 2014). The novel subclusters, *Ca*. Scalindua zhenghei and *Ca*. Scalindua pacifica, were tentatively proposed after being identified in the South China Sea (Hong et al., 2011a) and the Bohai Sea (Dang et al., 2013), respectively. *Ca*. Scalindua showed strong connections within its cluster but relatively low connectivity to other known anammox clusters (Figure 5). This observation reflected the microdiversity within *Ca*. Scalindua cluster. However, co-occurrence of *Ca*. Scalindua and OTUs from the unknown cluster was high and consistent, reflecting the close relationship between the two. The unknown cluster might be a second dominant cluster found in marine environments that has yet to be assigned to a genus-level designation.

In contrast to marine environments, freshwater environments showed a high diversity of anammox bacteria. The coexistence of *Ca*. Brocadia with known and unknown anammox clusters was generally found in previously reported freshwater habitats (Zhang et al., 2007; Hamersley et al., 2009; Hirsch et al., 2011; Yoshinaga et al., 2011; Hu et al., 2012b; Sonthiphand and Neufeld, 2013). However, one dominant anammox phylotype, *Ca*. Brocadia, was detected in the sediments of the Dongjiang River, Hong Kong (Sun et al., 2014), Lake Taihu, China (Wu et al., 2012), and the Grand River, Canada (Sonthiphand et al., 2013). Network analysis also showed that *Ca*. Brocadia clusters connected to OTUs from all known genera and the unknown cluster (Figure 5). *Ca*. Scalindua was solely detected in Lake Tanganyika, which is meromictic with a sharp chemocline (Schubert et al., 2006). Overall, *Ca*. Brocadia OTUs were found in all previously reported freshwater habitats, except Lake Tanganyika.

As with other freshwater environments, *Ca*. Brocadia was the major anammox phylotype detected in contaminated groundwater. However, *Ca*. Kuenenia, *Ca*. Jettenia, *Ca*. Scalindua, and OTUs from the unknown cluster were also present (Moore et al., 2011). However, most of sequences from this study were removed from this current analysis, resulting in low diversity richness and underestimation of anammox phylotypes in groundwater. There is insufficient groundwater-specific information due to a paucity of anammox groundwater surveys to date. We recommend further surveys of ammonia-rich groundwater isolation sources for obtaining a better understanding of anammox bacterial diversity in in this important low-oxygen and N-rich habitat.

The transitional zone between freshwater and marine environments, including estuary and mangrove sediment, is a dynamic habitat. River–sea interactions (i.e., river runoff, ocean tides, and inflow/outflow) possibly enhance the diversity of anammox bacteria. The mixture of known and unknown anammox clusters was evident in estuary habitats (Dale et al., 2009; Hirsch et al., 2011; Hu et al., 2012a; Hou et al., 2013) and mangrove sediment (Han and Gu, 2013; Li and Gu, 2013; Wang et al., 2013).

The combination of anammox OTUs associated with *Ca*. Brocadia, *Ca*. Kuenenia, *Ca*. Anammoxoglobus, and *Ca*. Jettenia was also found in various soil types, including peat soil (Hu et al., 2011), fertilized paddy soil (Zhu et al., 2011), a flooded paddy soil (Hu et al., 2013), and an agricultural soil (Shen et al., 2013). However, a single anammox phylotype was reported in some other soil types. *Ca*. Jettenia was recovered from manure pond soil (Sher et al., 2012) and permafrost soil (Humbert et al., 2010). *Ca*. Kuenenia was also detected in rhizosphere soil (Humbert et al., 2010). Interestingly, a rice paddy soil was dominated by *Ca*. Scalindua (Wang and Gu, 2013). The difference in soil properties (i.e., nutrients, O_2_, and pH) and depth reflected a microniche of anammox bacteria within terrestrial habitats (Zhu et al., 2011; Sher et al., 2012).

Our findings revealed the global distributions and diversities of anammox bacteria. These results added to previous knowledge about the geographical distributions and abundances of anammox bacteria in various environments, including marine (Dang et al., 2013; Shao et al., 2014), estuary (Hu et al., 2012a; Hou et al., 2013), soil (Sher et al., 2012), and freshwater (Sonthiphand and Neufeld, 2013; Sun et al., 2014). The abundances of anammox bacteria in marine sediments were positively correlated with marine water depth (Jaeschke et al., 2010; Sokoll et al., 2012; Trimmer et al., 2013; Shao et al., 2014). Low temperature likely favored the abundance of anammox bacteria in marine sediments (Russ et al., 2013). Consequently, anammox bacteria likely play a key role in the deep sea, where temperature is usually low (Jaeschke et al., 2010; Shao et al., 2014). In contrast to marine environments, the abundance of anammox bacteria showed a negative correlation with soil depth (Sher et al., 2012). The suggested reason for higher anammox bacterial abundance in surface soils was higher nutrient availability in upper layers compared to bottom layers of the soil profile. Substrate availability (NO^−^_2_ and NH^+^_4_) influenced anammox bacterial abundance in marine (Dang et al., 2010), estuary (Hou et al., 2013), freshwater (Wu et al., 2012; Sun et al., 2014), and soil (Shen et al., 2013) environments. Because NO^−^_2_ can be generated from NO^−^_3_ reduction, NO^−^_3_ concentration also affected the anammox bacterial abundance in estuary sediments (Hu et al., 2012a) and marine sediments (Han and Gu, 2013). In additional to quantifying the abundance of anammox bacteria, their activity must still be assessed in many of the above habitats to better understand their contributions to ecosystem N loss as part of the global N cycle.

## Concluding remarks

The global distribution pattern of anammox bacteria is controlled primarily by salinity. Distinct partitioning of anammox bacterial communities among natural and engineered ecosystems was also observed in our sequence survey. Insufficient information on anammox genomes and physiological properties is available to draw conclusions on how extrinsic factors (i.e., salinity, NH^+^_4_, NO^−^_2_) affect possible anammox bacterial mechanisms. More additional metagenomic studies of other anammox species will help compare and contrast the specific genes and their functions that influence the distribution and co-occurrence of anammox bacteria. Further investigations on kinetic and biochemical properties of more anammox species are needed to better understand the ecological niche partitioning of anammox bacteria. Freshwater is a promising habitat in which to discover novel anammox species and groundwater, in particular, may be an ideal study habitat for discovering anammox bacterial contributions to N loss in freshwater-related environments. Multidisciplinary approaches, including both metagenomic studies and molecular anammox surveys, are needed to fill in missing knowledge gaps.

### Conflict of interest statement

The authors declare that the research was conducted in the absence of any commercial or financial relationships that could be construed as a potential conflict of interest.

## References

[B1] Abma W. Schultz C. Mulder J. van der Star W. R. Strous M. Tokutomi T. (2007). Full-scale granular sludge anammox process. Water Sci. Technol. 55, 27–33 10.2166/wst.2007.238 17546966

[B2] Arrigo K. R. (2005). Marine microorganisms and global nutrient cycles. Nature 437, 349–355 10.1038/nature04159 16163345

[B3] Auguet J. C. Barberan A. Casamayor E. O. (2010). Global ecological patterns in uncultured Archaea. ISME J. 4, 182–190 10.1038/ismej.2009.109 19847207

[B4] Babbin A. R. Keil R. G. Devol A. H. Ward B. B. (2014). Organic matter stoichiometry, flux, and oxygen control nitrogen loss in the ocean. Science 344, 406–408 10.1126/science.1248364 24763588

[B5] Barberán A. Bates S. T. Casamayor E. O. Fierer N. (2012). Using network analysis to explore co-occurrence patterns in soil microbial communities. ISME J. 6, 343–351 10.1038/ismej.2011.119 21900968 PMC3260507

[B6] Bastian M. Heymann S. Jacomy M. (2009). Gephi: An open source software for exploring and manipulating networks, in International AAAI Conference on Weblogs and Social Media (San Jose, CA).

[B7] Biller S. J. Mosier A. C. Wells G. F. Francis C. A. (2012). Global biodiversity of aquatic ammonia-oxidizing archaea is partitioned by habitat. Front. Microbiol. 3:252 10.3389/fmicb.2012.00252 22826704 PMC3399221

[B8] Brosius J. Palmer M. L. Kennedy P. J. Noller H. F. (1978). Complete nucleotide sequence of a 16S ribosomal RNA gene from *Escherichia coli*. Proc. Natl. Acad. Sci. U.S.A. 75, 4801–4805 10.1073/pnas.75.10.4801 368799 PMC336208

[B9] Byrne N. Strous M. Crépeau V. Kartal B. Birrien J. L. Schmid M. (2009). Presence and activity of anaerobic ammonium-oxidizing bacteria at deep-sea hydrothermal vents. ISME J. 3, 117–123 10.1038/ismej.2008.72 18670398

[B10] Caporaso J. G. Kuczynski J. Stombaugh J. Bittinger K. Bushman F. D. Costello E. K. (2010). QIIME allows analysis of high-throughput community sequencing data. Nat. Methods 7, 335–336 10.1038/nmeth.f.303 20383131 PMC3156573

[B11] Casamayor E. O. Massana R. Benlloch S. Øvreås L. Díez B. Goddard V. J. (2002). Changes in archaeal, bacterial and eukaryal assemblages along a salinity gradient by comparison of genetic fingerprinting methods in a multipond solar saltern. Environ. Microbiol. 4, 338–348 10.1046/j.1462-2920.2002.00297.x 12071979

[B12] Castro-González M. Molina V. Rodríguez−Rubio E. Ulloa O. (2014). The first report of a microdiverse anammox bacteria community in waters of Colombian pacific, a transition area between prominent oxygen minimum zones of the eastern tropical pacific. Environ. Microbiol. Rep. [Epub ahead of print]. 10.1111/1758-2229.12165 25756112

[B13] Crump B. C. Hopkinson C. S. Sogin M. L. Hobbie J. E. (2004). Microbial biogeography along an estuarine salinity gradient: combined influences of bacterial growth and residence time. Appl. Environ. Microbiol. 70, 1494–1505 10.1128/AEM.70.3.1494-1505.2004 15006771 PMC365029

[B14] Dale O. R. Tobias C. R. Song B. (2009). Biogeographical distribution of diverse anaerobic ammonium oxidizing (anammox) bacteria in Cape Fear River Estuary. Environ. Microbiol. 11, 1194–1207 10.1111/j.1462-2920.2008.01850.x 19161435

[B15] Dalsgaard T. Thamdrup B. Farías L. Revsbech N. P. (2012). Anammox and dentrification in the oxygen minimum zone of the eastern South Pacific. Limnol. Oceanogr. 57, 1331–1346 10.4319/lo.2012.57.5.1331

[B16] Dang H. Chen R. Wang L. Guo L. Chen P. Tang Z. (2010). Environmental factors shape sediment anammox bacterial communities in hypernutrified Jiaozhou Bay, China. Appl. Environ. Microbiol. 76, 7036–7047 10.1128/AEM.01264-10 20833786 PMC2976235

[B17] Dang H. Zhou H. Zhang Z. Yu Z. Hua E. Liu X. (2013). Molecular detection of *Candidatus* Scalindua pacifica and environmental responses of sediment anammox bacterial community in the Bohai Sea, China. PLoS ONE 8:e61330 10.1371/journal.pone.0061330 23577216 PMC3620062

[B18] DeSantis T. Z. Hugenholtz P. Larsen N. Rojas M. Brodie E. L. Keller K. (2006). Greengenes, a chimera-checked 16S rRNA gene database and workbench compatible with ARB. Appl. Environ. Microbiol. 72, 5069–5072 10.1128/AEM.03006-05 16820507 PMC1489311

[B19] Edgar R. C. (2004). MUSCLE: multiple sequence alignment with high accuracy and high throughput. Nucleic Acids Res. 32, 1792–1797 10.1093/nar/gkh340 15034147 PMC390337

[B20] Egli K. Fanger U. Alvarez P. J. J. Siegrist H. van der Meer J. R. Zehnder A. J. B. (2001). Enrichment and characterization of an anammox bacterium from a rotating biological contactor treating ammonium-rich leachate. Arch. Microbiol. 175, 198–207 10.1007/s002030100255 11357512

[B21] Francis C. A. Beman J. M. Kuypers M. M. M. (2007). New processes and players in the nitrogen cycle: the microbial ecology of anaerobic and archaeal ammonia oxidation. ISME J. 1, 19–27 10.1038/ismej.2007.8 18043610

[B22] Francis C. A. O'Mullan G. D. Ward B. B. (2003). Diversity of ammonia monooxygenase (*amoA*) genes across environmental gradients in Chesapeake Bay sediments. Geobiology 1, 129–140 10.1046/j.1472-4669.2003.00010.x

[B23] Fu L. Niu B. Zhu Z. Wu S. Li W. (2012). CD-HIT: accelerated for clustering the next-generation sequencing data. Bioinformatics 28, 3150–3152 10.1093/bioinformatics/bts565 23060610 PMC3516142

[B24] Fuchsman C. A. Staley J. T. Oakley B. B. Kirkpatrick J. B. Murray J. W. (2012). Free-living and aggregate-associated *Planctomycetes* in the Black Sea. FEMS Microbiol. Ecol. 80, 402–416 10.1111/j.1574-6941.2012.01306.x 22251018

[B25] Galtier N. Gouy M. Gautier C. (1996). SEAVIEW and PHYLO_WIN: two graphic tools for sequence alignment and molecular phylogeny. Bioinformatics 12, 543–548 10.1093/bioinformatics/12.6.543 9021275

[B26] Gori F. Tringe S. G. Kartal B. Marchiori E. Machiori E. Jetten M. S. M. (2011). The metagenomic basis of anammox metabolism in *Candidatus* ‘Brocadia fulgida.’ Biochem. Soc. Trans. 39, 1799–1804 10.1042/BST20110707 22103529

[B27] Guindon S. Gascuel O. (2003). A simple, fast, and accurate algorithm to estimate large phylogenies by maximum likelihood. Syst. Biol. 52, 696–704 10.1080/10635150390235520 14530136

[B28] Hamersley M. R. Woebken D. Boehrer B. Schultze M. Lavik G. Kuypers M. M. M. (2009). Water column anammox and denitrification in a temperate permanently stratified lake (Lake Rassnitzer, Germany). Syst. Appl. Microbiol. 32, 571–582 10.1016/j.syapm.2009.07.009 19716251

[B29] Han P. Gu J. D. (2013). More refined diversity of anammox bacteria recovered and distribution in different ecosystems. Appl. Microbiol. Biotechnol. 97, 3653–3663 10.1007/s00253-013-4756-6 23515834

[B30] Hirsch M. D. Long Z. T. Song B. (2011). Anammox bacterial diversity in various aquatic ecosystems based on the detection of hydrazine oxidase genes (*hzoA/hzoB*). Microb. Ecol. 61, 264–276 10.1007/s00248-010-9743-1 20838786

[B31] Hong Y. G. Li M. Cao H. Gu J. D. (2011a). Residence of habitat-specific anammox bacteria in the deep-sea subsurface sediments of the South China Sea: analyses of marker gene abundance with physical chemical parameters. Microb. Ecol. 62, 36–47 10.1007/s00248-011-9849-0 21491114 PMC3141849

[B32] Hong Y. G. Yin B. Zheng T. L. (2011b). Diversity and abundance of anammox bacterial community in the deep-ocean surface sediment from equatorial Pacific. Appl. Microbiol. Biotechnol. 89, 1233–1241 10.1007/s00253-010-2925-4 20949269

[B33] Hou L. Zheng Y. Liu M. Gong J. Zhang X. Yin G. (2013). Anaerobic ammonium oxidation (anammox) bacterial diversity, abundance, and activity in marsh sediments of the Yangtze Estuary. J. Geophys. Res. 118, 1237–1246 10.1002/jgrg.20108

[B34] Hu B. L. Rush D. van der Biezen E. Zheng P. van Mullekom M. Schouten S. (2011). New anaerobic, ammonium-oxidizing community enriched from peat soil. Appl. Environ. Microbiol. 77, 966–971 10.1128/AEM.02402-10 21148690 PMC3028707

[B35] Hu B. L. Shen L. D. Du P. Zheng P. Xu X. Zeng J. (2012a). The influence of intense chemical pollution on the community composition, diversity and abundance of anammox bacteria in the Jiaojiang Estuary (China). PLoS ONE 7:e33826 10.1371/journal.pone.0033826 22470481 PMC3309935

[B36] Hu B. L. Shen L. D. Liu S. Cai C. Chen T. T. Kartal B. (2013). Enrichment of an anammox bacterial community from a flooded paddy soil. Environ. Microbiol. Rep. 5, 483–489 10.1111/1758-2229.12038 23754729

[B37] Hu B. L. Shen L. D. Zheng P. Hu A. H. Chen T. T. Cai C. (2012b). Distribution and diversity of anaerobic ammonium-oxidizing bacteria in the sediments of the Qiantang River. Environ. Microbiol. Rep. 4, 540–547 10.1111/j.1758-2229.2012.00360.x 23760899

[B38] Hu B. L. Zheng P. Tang C. J. Chen J. W. van der Biezen E. Zhang L. (2010). Identification and quantification of anammox bacteria in eight nitrogen removal reactors. Water Res. 44, 5014–5020 10.1016/j.watres.2010.07.021 20705314

[B39] Hu Z. Speth D. R. Francoijs K. J. Quan Z. X. Jetten M. S. M. (2012c). Metagenome analysis of a complex community reveals the metabolic blueprint of anammox bacterium “*Candidatus* Jettenia asiatica.” Front. Microbiol. 3:366 10.3389/fmicb.2012.00366 23112795 PMC3482989

[B40] Humbert S. Tarnawski S. Fromin N. Mallet M. P. Aragno M. Zopfi J. (2010). Molecular detection of anammox bacteria in terrestrial ecosystems: distribution and diversity. ISME J. 4, 450–454 10.1038/ismej.2009.125 20010634

[B41] Jaeschke A. Abbas B. Zabel M. Hopmans E. C. Schouten S. Sinninghe Damsté J. S. (2010). Molecular evidence for anaerobic ammonium-oxidizing (anammox) bacteria in continental shelf and slope sediments off northwest Africa. Limnol. Oceanogr. 55, 365–376 10.4319/lo.2010.55.1.0365

[B42] Jensen M. M. Lam P. Revsbech N. P. Nagel B. Gaye B. Jetten M. S. M. (2011). Intensive nitrogen loss over the Omani Shelf due to anammox coupled with dissimilatory nitrite reduction to ammonium. ISME J. 5, 1660–1670 10.1038/ismej.2011.44 21509044 PMC3176517

[B43] Jetten M. S. M. Cirpus I. Kartal B. van Niftrik L. A. van de Pas-Schoonen K. T. Sliekers O. (2005). 1994–2004: 10 years of research on the anaerobic oxidation of ammonium. Biochem. Soc. Trans. 33, 119–123 10.1042/BST0330119 15667281

[B44] Jetten M. S. M. Logemann S. Muyzer G. Robertson L. A. de Vries S. van Loosdrecht M. C. M. (1997). Novel principles in the microbial conversion of nitrogen compounds. Anton. Leeuw. 71, 75–93 10.1023/A:1000150219937 9049020

[B45] Kartal B. Koleva M. Arsov R. van der Star W. R. Jetten M. S. M. Strous M. (2006). Adaptation of a freshwater anammox population to high salinity wastewater. J. Biotechnol. 126, 546–553 10.1016/j.jbiotec.2006.05.012 16806555

[B46] Kartal B. Rattray J. van Niftrik L. A. van de Vossenberg J. Schmid M. C. Webb R. I. (2007). *Candidatus* “Anammoxoglobus propionicus” a new propionate oxidizing species of anaerobic ammonium oxidizing bacteria. Syst. Appl. Microbiol. 30, 39–49 10.1016/j.syapm.2006.03.004 16644170

[B47] Kartal B. van Niftrik L. A. Rattray J. van de Vossenberg J. Schmid M. C. Sinninghe Damsté J. S. (2008). *Candidatus* “Brocadia fulgida”: an autofluorescent anaerobic ammonium oxidizing bacterium. FEMS Microbiol. Ecol. 63, 46–55 10.1111/j.1574-6941.2007.00408.x 18081590

[B48] Koop-Jakobsen K. Giblin A. E. (2009). Anammox in tidal marsh sediments: the role of salinity, nitrogen loading, and marsh vegetation. Estuaries Coasts 32, 238–245 10.1007/s12237-008-9131-y

[B49] Kuenen J. G. Jetten M. S. M. (2001). Extraordinary anaerobic ammonium oxidizing bacteria. Am. Soc. Microbiol. News 67, 456–463

[B50] Kuypers M. M. M. Lavik G. Woebken D. Schmid M. C. Fuchs B. M. Amann R. (2005). Massive nitrogen loss from the Benguela upwelling system through anaerobic ammonium oxidation. Proc. Natl. Acad. Sci. U.S.A. 102, 6478–6483 10.1073/pnas.0502088102 15843458 PMC556276

[B51] Kuypers M. M. M. Sliekers A. O. Lavik G. Schmid M. C. Jørgensen B. B. Kuenen J. G. (2003). Anaerobic ammonium oxidation by anammox bacteria in the Black Sea. Nature 422, 608–611 10.1038/nature01472 12686999

[B52] Lam P. Kuypers M. M. M. (2011). Microbial nitrogen cycling processes in oxygen minimum zones. Annu. Rev. Marine Sci. 3, 317–345 10.1146/annurev-marine-120709-142814 21329208

[B53] Li H. Chen S. Mu B. Z. Gu J. D. (2010). Molecular detection of anaerobic ammonium-oxidizing (anammox) bacteria in high-temperature petroleum reservoirs. Microb. Ecol. 60, 771–783 10.1007/s00248-010-9733-3 20740282 PMC2974184

[B54] Li M. Gu J. D. (2013). Community structure and transcript responses of anammox bacteria, AOA, and AOB in mangrove sediment microcosms amended with ammonium and nitrite. Appl. Microbiol. Biotechnol. 97, 9859–9874 10.1007/s00253-012-4683-y 23455621

[B55] Li M. Hong Y. Cao H. Gu J. D. (2013). Community structures and distribution of anaerobic ammonium oxidizing and *nirS*-encoding nitrite-reducing bacteria in surface sediments of the South China Sea. Microb. Ecol. 66, 281–296 10.1007/s00248-012-0175-y 23354291

[B56] Li M. Hong Y. G. Cao H. L. Gu J. D. (2011). Mangrove trees affect the community structure and distribution of anammox bacteria at an anthropogenic-polluted mangrove in the Pearl River Delta reflected by 16S rRNA and hydrazine oxidoreductase (HZO) encoding gene analyses. Ecotoxicology 20, 1780–1790 10.1007/s10646-011-0711-4 21735127 PMC3195777

[B57] Lozupone C. A. Knight R. (2005). UniFrac: a new phylogenetic method for comparing microbial communities. Appl. Environ. Microbiol. 71, 8228–8235 10.1128/AEM.71.12.8228-8235.2005 16332807 PMC1317376

[B58] Lozupone C. A. Knight R. (2007). Global patterns in bacterial diversity. Proc. Natl. Acad. Sci. U.S.A. 104, 11436–11440 10.1073/pnas.0611525104 17592124 PMC2040916

[B59] Lynch M. D. Masella A. P. Hall M. W. Bartram A. K. Neufeld J. D. (2013). AXIOME: automated exploration of microbial diversity. GigaScience 2:3 10.1186/2047-217X-2-3 23587322 PMC3626533

[B60] Moore T. A. Xing Y. Lazenby B. Lynch M. D. Schiff S. Robertson W. D. (2011). Prevalence of anaerobic ammonium-oxidizing bacteria in contaminated groundwater. Environ. Sci. Technol. 45, 7217–7225 10.1021/es201243t 21786759

[B61] Mulder A. van de Graaf A. A. Robertson L. A. Kuenen J. G. (1995). Anaerobic ammonium oxidation discovered in a denitrifying fluidized bed reactor. FEMS Microbiol. Ecol. 16, 177–183 10.1111/j.1574-6941.1995.tb00281.x

[B62] Nakajima J. Sakka M. Kimura T. Furukawa K. Sakka K. (2008). Enrichment of anammox bacteria from marine environment for the construction of a bioremediation reactor. Appl. Microbiol. Biotechnol. 77, 1159–1166 10.1007/s00253-007-1247-7 17965857

[B63] Nawrocki E. P. Eddy S. R. (2013). Infernal 1.1: 100-fold faster RNA homology searches. Bioinformatics 29, 2933–2935 10.1093/bioinformatics/btt509 24008419 PMC3810854

[B64] Oksanen J. Kindt R. Legendre P. O'Hara B. Simpson G. L. Solymos P. (2008). Vegan: community ecology package. R package version 1:8

[B65] Osaka T. Kimura Y. Otsubo Y. Suwa Y. Tsuneda S. Isaka K. (2012). Temperature dependence for anammox bacteria enriched from freshwater sediments. J. Biosci. Bioeng. 114, 429–434 10.1016/j.jbiosc.2012.05.003 22652085

[B66] Oshiki M. Shimokawa M. Fujii N. Satoh H. Okabe S. (2011). Physiological characteristics of the anaerobic ammonium-oxidizing bacterium “*Candidatus* Brocadia sinica.” Microbiology 157, 1706–1713 10.1099/mic.0.048595-0 21474538

[B67] Park H. Rosenthal A. Jezek R. Ramalingam K. Fillos J. Chandran K. (2010). Impact of inocula and growth mode on the molecular microbial ecology of anaerobic ammonia oxidation (anammox) bioreactor communities. Water Res. 44, 5005–5013 10.1016/j.watres.2010.07.022 20684970

[B68] Pollet T. Tadonléké R. D. Humbert J. F. (2011). Comparison of primer sets for the study of *Planctomycetes* communities in lentic freshwater ecosystems. Environ. Microbiol. Rep. 3, 254–261 10.1111/j.1758-2229.2010.00219.x 23761258

[B69] Quan Z. X. Rhee S. K. Zuo J. E. Yang Y. Bae J. W. Park J. R. (2008). Diversity of ammonium-oxidizing bacteria in a granular sludge anaerobic ammonium-oxidizing (anammox) reactor. Environ. Microbiol. 10, 3130–3139 10.1111/j.1462-2920.2008.01642.x 18479446

[B70] R Core Team (2013). R: A Language and Environment for Statistical Computing. Vienna: R Foundation for Statistical Computing

[B71] Rich J. J. Dale O. R. Song B. Ward B. B. (2008). Anaerobic ammonium oxidation (anammox) in Chesapeake Bay sediments. Microb. Ecol. 55, 311–320 10.1007/s00248-007-9277-3 17619213

[B72] Rothrock M. J. Vanotti M. B. Szogi A. A. Gonzalez M. C. Fujii T. (2011). Long-term preservation of anammox bacteria. Appl. Microbiol. Biotechnol. 92, 147–157 10.1007/s00253-011-3316-1 21590289

[B73] Russ L. Kartal B. Op Den Camp H. J. M. Sollai M. Le Bruchec J. Caprais J. C. (2013). Presence and diversity of anammox bacteria in cold hydrocarbon-rich seeps and hydrothermal vent sediments of the Guaymas Basin. Front. Microbiol. 4:219 10.3389/fmicb.2013.00219 23935595 PMC3731535

[B74] Rysgaard S. Thastum P. Dalsgaard T. Christensen P. B. Sloth N. P. (1999). Effects of salinity on NH^+^_4_ adsorption capacity, nitrification, and denitrification in Danish estuarine sediments. Estuaries Coasts 22, 21–30 10.2307/1352923

[B75] Santoro A. E. Francis C. A. De Sieyes N. R. Boehm A. B. (2008). Shifts in the relative abundance of ammonia-oxidizing bacteria and archaea across physicochemical gradients in a subterranean estuary. Environ. Microbiol. 10, 1068–1079 10.1111/j.1462-2920.2007.01547.x 18266758

[B76] Schmid M. C. Risgaard-Petersen N. van de Vossenberg J. Kuypers M. M. M. Lavik G. Petersen J. (2007). Anaerobic ammonium-oxidizing bacteria in marine environments: widespread occurrence but low diversity. Environ. Microbiol. 9, 1476–1484 10.1111/j.1462-2920.2007.01266.x 17504485

[B77] Schmid M. C. Twachtmann U. Klein M. Strous M. Juretschko S. Jetten M. S. M. (2000). Molecular evidence for genus level diversity of bacteria capable of catalyzing anaerobic ammonium oxidation. Syst. Appl. Microbiol. 23, 93–106 10.1016/S0723-2020(00)80050-8 10879983

[B78] Schmid M. C. Walsh K. Webb R. Rijpstra W. I. van de Pas-Schoonen K. T. Verbruggen M. J. (2003). *Candidatus* “Scalindua brodae,” sp. nov., *Candidatus* “Scalindua wagneri,” sp. nov., two new species of anaerobic ammonium oxidizing bacteria. Syst. Appl. Microbiol. 26, 529–538 10.1078/072320203770865837 14666981

[B79] Schubert C. J. Durisch-Kaiser E. Wehrli B. Lam P. Kuypers M. M. M. (2006). Anaerobic ammonium oxidation in a tropical freshwater system (Lake Tanganyika). Environ. Microbiol. 8, 1857–1863 10.1111/j.1462-2920.2006.01074.x 16958766

[B80] Shao S. Luan X. Dang H. Zhou H. Zhao Y. Liu H. (2014). Deep-sea methane seep sediments in the Okhotsk Sea sustain diverse and abundant anammox bacteria. FEMS Microbiol. Ecol. 87, 503–516 10.1111/1574-6941.12241 24164560

[B81] Shen L. D. Liu S. Lou L. P. Liu W. P. Xu X. Y. Zheng P. (2013). Broad distribution of diverse anaerobic ammonium-oxidizing bacteria in Chinese agricultural soils. Appl. Environ. Microbiol. 79, 6167–6172 10.1128/AEM.00884-13 23747706 PMC3811364

[B82] Sher Y. Baram S. Dahan O. Ronen Z. Nejidat A. (2012). Ammonia transformations and abundance of ammonia oxidizers in a clay soil underlying a manure pond. FEMS Microbiol. Ecol. 81, 145–155 10.1111/j.1574-6941.2012.01347.x 22385337

[B83] Sokoll S. Holtappels M. Lam P. Collins G. Schlüter M. Lavik G. (2012). Benthic nitrogen loss in the Arabian Sea off Pakistan. Front. Microbiol. 3:395 10.3389/fmicb.2012.00395 23226143 PMC3508403

[B84] Sonthiphand P. Cejudo E. Schiff S. L. Neufeld J. D. (2013). Wastewater effluent impacts ammonia-oxidizing prokaryotes of the Grand River, Canada. Appl. Environ. Microbiol. 79, 7454–7465 10.1128/AEM.02202-13 24056472 PMC3837731

[B85] Sonthiphand P. Neufeld J. D. (2013). Evaluating primers for profiling anaerobic ammonia oxidizing bacteria within freshwater environments. PLoS ONE 8:e57242 10.1371/journal.pone.0057242 23505422 PMC3591393

[B86] Speth D. R. Hu B. Bosch N. Keltjens J. T. Stunnenberg H. G. Jetten M. S. M. (2012). Comparative genomics of two independently enriched “*Candidatus* Kuenenia stuttgartiensis” anammox bacteria. Front. Microbiol. 3:307 10.3389/fmicb.2012.00307 22934093 PMC3423927

[B87] Strous M. Heijnen J. Kuenen J. G. Jetten M. S. M. (1998). The sequencing batch reactor as a powerful tool for the study of slowly growing anaerobic ammonium-oxidizing microorganisms. Appl. Microbiol. Biotechnol. 50, 589–596 10.1007/s002530051340

[B88] Strous M. Kuenen J. G. Jetten M. S. M. (1999). Key physiology of anaerobic ammonium oxidation. Appl. Environ. Microbiol. 65, 3248–3250 10388731 10.1128/aem.65.7.3248-3250.1999 PMC91484

[B89] Strous M. Pelletier E. Mangenot S. Rattei T. Lehner A. Taylor M. W. (2006). Deciphering the evolution and metabolism of an anammox bacterium from a community genome. Nature 440, 790–794 10.1038/nature04647 16598256

[B90] Sun W. Xu M. Y. Wu W. M. Guo J. Xia C. Y. Sun G. P. (2014). Molecular diversity and distribution of anammox community in sediments of the Dongjiang River, a drinking water source of Hong Kong. J. Appl. Microbiol. 116, 464–476 10.1111/jam.12367 24125160

[B91] Third K. Sliekers A. O. Kuenen J. G. Jetten M. S. M. (2001). The CANON system (completely autotrophic nitrogen-removal over nitrite) under ammonium limitation: interaction and competition between three groups of bacteria. Syst. Appl. Microbiol. 24, 588–596 10.1078/0723-2020-00077 11876366

[B92] Trimmer M. Engström P. (2011). The environmental distribution, activity and ecology of anammox, in Nitrification, eds Ward B. B. Klotz M. G. Arp D. J. (Washington, DC: ASM Press), 201–235

[B93] Trimmer M. Engström P. Thamdrup B. (2013). Stark contrast in denitrification and anammox across the deep Norwegian trench in the Skagerrak. Appl. Environ. Microbiol. 79, 7381–7389 10.1128/AEM.01970-13 24056465 PMC3837768

[B94] Trimmer M. Nicholls J. C. Deflandre B. (2003). Anaerobic ammonium oxidation measured in sediments along the Thames estuary, United Kingdom. Appl. Environ. Microbiol. 69, 6447–6454 10.1128/AEM.69.11.6447-6454.2003 14602599 PMC262292

[B95] van de Graaf A. A. Mulder A. de Bruijn P. Jetten M. S. M. Robertson L. A. Kuenen J. G. (1995). Anaerobic oxidation of ammonium is a biologically mediated process. Appl. Environ. Microbiol. 61, 1246–1251 7747947 10.1128/aem.61.4.1246-1251.1995 PMC167380

[B96] van der Star W. R. Abma W. R. Blommers D. Mulder J. W. Tokutomi T. Strous M. (2007). Startup of reactors for anoxic ammonium oxidation: experiences from the first full-scale anammox reactor in Rotterdam. Water Res. 41, 4149–4163 10.1016/j.watres.2007.03.044 17583763

[B97] van der Star W. R. Miclea A. I. van Dongen U. G. Muyzer G. Picioreanu C. van Loosdrecht M. C. M. (2008a). The membrane bioreactor: a novel tool to grow anammox bacteria as free cells. Biotechnol. Bioeng. 101, 286–294 10.1002/bit.21891 18421799

[B98] van der Star W. R. van de Graaf M. J. Kartal B. Picioreanu C. Jetten M. S. M. van Loosdrecht M. C. M. (2008b). Response of anaerobic ammonium-oxidizing bacteria to hydroxylamine. Appl. Environ. Microbiol. 74, 4417–4426 10.1128/AEM.00042-08 18515490 PMC2493174

[B99] van de Vossenberg J. Rattray J. E. Geerts W. Kartal B. van Niftrik L. A. van Donselaar E. G. (2008). Enrichment and characterization of marine anammox bacteria associated with global nitrogen gas production. Environ. Microbiol. 10, 3120–3129 10.1111/j.1462-2920.2008.01643.x 18462401

[B100] van de Vossenberg J. Woebken D. Maalcke W. J. Wessels H. J. Dutilh B. E. Kartal B. (2013). The metagenome of the marine anammox bacterium ‘*Candidatus* Scalindua profunda' illustrates the versatility of this globally important nitrogen cycle bacterium. Environ. Microbiol. 15, 1275–1289 10.1111/j.1462-2920.2012.02774.x 22568606 PMC3655542

[B101] van Dongen U. Jetten M. S. M. van Loosdrecht M. C. M. (2001). The SHARON-Anammox process for treatment of ammonium rich wastewater. Water Sci. Technol. 44, 153–160 11496667

[B102] Villanueva L. Speth D. Vanalen T. Hoischen A. Jetten M. S. M. (2014). Shotgun metagenomic data reveals signifcant abundance but low diversity of “*Candidatus* Scalindua” marine anammox bacteria in the Arabian Sea oxygen minimum zone. Front. Microbiol. 5:31 10.3389/fmicb.2014.00031 24550902 PMC3913995

[B103] Wang J. Gu J. D. (2013). Dominance of *Candidatus* Scalindua species in anammox community revealed in soils with different duration of rice paddy cultivation in Northeast China. Appl. Microbiol. Biotechnol. 97, 1785–1798 10.1007/s00253-012-4036-x 22526793 PMC3562551

[B104] Wang Y. F. Feng Y. Y. Ma X. Gu J. D. (2013). Seasonal dynamics of ammonia/ammonium-oxidizing prokaryotes in oxic and anoxic wetland sediments of subtropical coastal mangrove. Appl. Microbiol. Biotechnol. 97, 7919–7934 10.1007/s00253-012-4510-5 23099914 PMC3745829

[B105] Woebken D. Lam P. Kuypers M. M. M. Naqvi S. W. Kartal B. Strous M. (2008). A microdiversity study of anammox bacteria reveals a novel *Candidatus* Scalindua phylotype in marine oxygen minimum zones. Environ. Microbiol. 10, 3106–3119 10.1111/j.1462-2920.2008.01640.x 18510553

[B106] Wu Y. Xiang Y. Wang J. Wu Q. L. (2012). Molecular detection of novel anammox bacterial clusters in the sediments of the shallow freshwater Lake Taihu. Geomicrobiol. J. 29, 852–859 10.1080/01490451.2011.635760

[B107] Yoshie S. Noda N. Tsuneda S. Hirata A. Inamori Y. (2004). Salinity decreases nitrite reductase gene diversity in denitrifying bacteria of wastewater treatment systems. Appl. Environ. Microbiol. 70, 3152–3157 10.1128/AEM.70.5.3152-3157.2004 15128582 PMC404418

[B108] Yoshinaga I. Amano T. Yamagishi T. Okada K. Ueda S. Sako Y. (2011). Distribution and diversity of anaerobic ammonium oxidation (anammox) bacteria in the sediment of a eutrophic freshwater lake, Lake Kitaura, Japan. Microbes Environ. 26, 189–197 10.1264/jsme2.ME10184 21558678

[B109] Zhang Y. Ruan X. H. Op den Camp H. J. M. Smits T. J. M. Jetten M. S. M. Schmid M. C. (2007). Diversity and abundance of aerobic and anaerobic ammonium-oxidizing bacteria in freshwater sediments of the Xinyi River (China). Environ. Microbiol. 9, 2375–2382 10.1111/j.1462-2920.2007.01357.x 17686033

[B110] Zhu G. Wang S. Wang Y. Wang C. Risgaard-Petersen N. Jetten M. S. M. (2011). Anaerobic ammonia oxidation in a fertilized paddy soil. ISME J. 5, 1905–1912 10.1038/ismej.2011.63 21593796 PMC3223303

